# Updating the WHO target product profile for next-generation *Mycobacterium tuberculosis* drug susceptibility testing at peripheral centres

**DOI:** 10.1371/journal.pgph.0001754

**Published:** 2023-03-31

**Authors:** Emily Lai-Ho MacLean, Paolo Miotto, Lice González Angulo, Matteo Chiacchiaretta, Timothy M. Walker, Martina Casenghi, Camilla Rodrigues, Timothy C. Rodwell, Philip Supply, Emmanuel André, Mikashmi Kohli, Morten Ruhwald, Daniela Maria Cirillo, Nazir Ismail, Matteo Zignol

**Affiliations:** 1 Department of Epidemiology, Biostatistics and Occupational Health, McGill University, Montreal, Canada; 2 McGill International TB Centre, Montreal, Canada; 3 Emerging Bacterial Pathogens Unit, Division of Immunology, Transplantation and Infectious Diseases, IRCCS San Raffaele Scientific Institute, Milan, Italy; 4 Global TB Programme, World Health Organization, Geneva, Switzerland; 5 Oxford University Clinical Research Unit, Ho Chi Minh City, Vietnam; 6 Department of Innovation and New Technology, Elizabeth Glaser Paediatric AIDS Foundation, Geneva, Switzerland; 7 P. D. Hinduja National Hospital and Medical Research Centre, Mumbai, India; 8 FIND, Geneva, Switzerland; 9 Department of Medicine, University of California, San Diego, La Jolla, California, United States of America; 10 Univ. de Lille, CNRS, INSERM, CHU Lille; Institut Pasteur de Lille, U1019-UMR 9017-CIIL (Center for Infection and Immunity of Lille), Lille, France; 11 Laboratory of Clinical Bacteriology and Mycology, Dept of Microbiology and Immunology, KU Leuven, Leuven, Belgium; 12 Department of Laboratory Medicine, UZ Leuven Hospitals, Leuven, Belgium; Aga Khan University, PAKISTAN

## Abstract

There were approximately 10 million tuberculosis (TB) cases in 2020, of which 500,000 were drug-resistant. Only one third of drug-resistant TB cases were diagnosed and enrolled on appropriate treatment, an issue partly driven by a lack of rapid, accurate drug-susceptibility testing (DST) tools deployable in peripheral settings. In 2014, World Health Organization (WHO) published target product profiles (TPPs) which detailed minimal and optimal criteria to address high-priority TB diagnostic needs, including DST. Since then, the TB community’s needs have evolved; new treatment regimens, changes in TB definitions, further emergence of drug resistance, technological advances, and changing end-users requirements have necessitated an update. The DST TPP’s revision was therefore undertaken by WHO with the Stop TB Partnership New Diagnostics Working Group. We describe the process of updating the TPP for next-generation TB DST for use at peripheral centres, highlight key updates, and discuss guidance regarding technical and operational specifications.

## Introduction

Although substantial progress toward ending tuberculosis (TB) has been attained in recent years, considerable gaps remain in achieving global targets. These gaps have been exacerbated by the COVID-19 pandemic. Nearly 10 million people are estimated to develop TB each year. However, in 2020, only 5.8 million cases were notified worldwide [1], of which 4.8 million were diagnosed with pulmonary TB. Three million were bacteriologically confirmed, and only 2.1 million were tested for rifampicin (RIF) resistance [1]. Furthermore, nearly half a million people developed rifampicin-resistant TB (RR-TB) in 2020; the majority of these cases were multi-drug resistant (MDR; defined as resistant at least to RIF and isoniazid [INH]) [1], and only 1 in 3 cases were notified and treated for MDR/RR-TB. Closing these gaps in the cascade of care requires a multi-faceted approach, including developing next-generation drug susceptibility testing (DST) technologies. Such tests should have improved accuracy, cover priority drugs, be easy to use, offer short turnaround times, and enable near-patient testing [2, 3].

Phenotypic DST remains the reference standard for detecting resistance to many anti-TB medicines, and although accurate for most drugs, access is limited due to infrastructure and technical skill requirements. Phenotypic DST is rarely available outside of reference laboratories or centralised levels of healthcare systems, and confirming mycobacterial growth and detecting drug resistance requires prolonged time–up to two months for full first-line and second-line resistance profiling. The resulting diagnostic delays impact timely treatment adaptation and may lead to inadequate treatment, thereby amplifying drug resistance. Rapid DST platforms endorsed by the World Health Organization (WHO) [4] have significantly reduced turnaround time from months to hours [5]. However, a limitation of such assays is that their development has primarily focused on detecting resistance to a single first-line drug–RIF. More recently, newer platforms have incorporated testing for INH and other medicines, but rapid detection of resistance for repurposed and some newer medicines is limited to next-generation sequencing-based tests (see below).

The WHO’s latest definitions of extensively drug-resistant (XDR) TB and pre-XDR-TB [6] in 2021 have further prioritised other key second-line drugs that are important in managing drug-resistant TB. XDR-TB is now defined as MDR/RR-TB that is also resistant to any fluoroquinolone (FQ) and at least one additional Group A drug (bedaquiline [BDQ] and linezolid [LZD]), while pre-XDR-TB is now defined as MDR/RR-TB that is also resistant to any FQ. Now more than ever, these changes and the evolving treatment pipeline underscore the need for better and faster DST methods for TB programmes and end-users.

The urgency of meeting the global TB elimination targets for 2035 combined with tackling the pressing threat of drug resistance requires redoubled efforts, innovation, and the ability to identify drug resistance early. Fortunately, recent years have seen advances in TB diagnostics pipeline, such as next-generation sequencing (NGS) [7, 8] or end-to-end tests for integrated TB and drug resistance detection [9], with some potentially deployable at peripheral levels of healthcare systems [10, 11]. The increased availability of diagnostic technologies that can support drug resistance detection at peripheral sites and are based on affordable, easy-to-adopt technologies is critical for timely triaging of patients and initiation of the most appropriate and effective treatment regimens.

In 2014, WHO published a series of high-priority target product profiles (TPPs) for a variety of TB diagnostic tests to be prioritised for investment, research and development [12]. These TPPs were developed to plan and better guide the development of ‘fit for purpose’ tests, detailing technical specifications critical to end-users. As diagnostic technologies and therapies have evolved in the last few years [13–15], the projected timeframe of 5 years to attain the 2014 high-priority products was reached; a revision and update of the TPP to address the current gaps was therefore needed. One of the TPPs titled “Next-generation drug-susceptibility testing at microscopy centres” is the base chapter for the current update.

In this paper, we draw attention to the prevailing and urgent needs for prioritising TB DST, with emphasis on the development of tools for use at peripheral centres as the TB treatment landscape and patient needs continue to evolve [16].

## Methods

This TPP largely summarises the specifications across four key domains: (i) scope and prioritisation of anti-TB agents for testing; (ii) analytical performance; (iii) operational and infrastructure requirements; and (iv) pricing; all considered in the context of feasibility, affordability and adoption of novel tools. To better identify and capture the desired characteristics for next-generation DST for peripheral healthcare facilities, the process centred around engagement and collaborative efforts involving key stakeholders and partners, including researchers, clinicians, TB advocates, members of civil society organisations and policy makers. To maximise the public health value of this TPP and accelerate research and development efforts, funding agencies and industry partners were also invited to share their experience and views on this process.

The TPP was updated in three steps − a Delphi-like process, public comment on the TPP document, and the final WHO stakeholders’ consultation meeting.

### Delphi-like process

A draft TPP document was prepared to promote discussion between the different groups of stakeholders. The working document was drafted in collaboration with the New Diagnostics Working Group’s NGS and Next-generation DST Task Force between April and December 2018. The draft was circulated for expert review in January and February 2019.

A Delphi-like process was utilised to gather critical comments and feedback on the working document in 2019. The working document and a survey questionnaire consisting of 47 specific questions were then shared with a broader audience through the New Diagnostics Working Group website and the Global Tuberculosis Network (for draft TPP, survey questions based on draft TPP, survey comments, and updated draft TPP see https://stoptb.org/wg/new_diagnostics/assets/documents/NDWG_TPP_microscopy%20centres_REPORT%20SURVEY%202019_ver21Jun2019.pdf). Additional working groups such as the Global Laboratory Initiative (GLI-TB) and the European Laboratory Initiative (ELI-TB) were also invited to respond to the Delphi-like survey. The survey was available online from 29 April to 24 May 2019. Participants were requested to express their level of agreement on the proposed characteristics that require updating; their agreement was measured using Likert scale ranging from 1 to 5 (1-disagree, 2-mostly disagree, 3-don’t agree or disagree, 4-mostly agree, 5-fully agree). In cases where stakeholders disagreed with any of the proposed updated desired characteristics, they also had the opportunity to provide comments. Agreement was reached if ≥80% of respondents scored 4 (Mostly agree) or 5 (Fully agree) for the content and wording of a particular characteristic. If so, the characteristic remained as originally proposed and progressed to the next step.

### Public comment

The TPP document was revised to integrate comments received through the Delphi process and then redistributed in 2019 through a public comment process (see S1 File for questions included in public comment survey). A second round of public comments were elicited to incorporate the perspectives of a broader audience; the revised document and an additional survey of 29 questions were published on the WHO website for public comment from 7 January 2021 to 7 February 2021. Individuals were asked to provide comments on any characteristic in the TPP document (as presented before or after public comment) with which they disagreed.

### WHO stakeholder consultation

Feedback received through the public comment process was presented and discussed at the virtual WHO stakeholder consultation in 2021. During this meeting, each characteristic of the TPP was presented as proposed, with dissenting comments accompanying the text to generate discussion. Consensus was sought for each characteristic via discussion and use of the virtual chat function to ask for agreement or disagreement at the end of a characteristic’s consideration.

### Assumptions

Specific requirements of the test were described by individual characteristics, and for each characteristic, ‘Optimal’ and ‘Minimal’ specifications were defined. ‘Optimal’ criteria provide the ideal output for a particular characteristic that is believed to be achievable; a test characteristic that is ‘Optimal’ would provide the greatest impact for end users, clinicians, and people affected by TB. ‘Minimal’ criteria describe the lowest acceptable output for a particular test characteristic, i.e., test products must meet the ‘Minimal’ criteria in order to be acceptable. In certain instances, a test may still be deemed acceptable if any shortcomings pertain to some targets, so long as criteria for the most critical characteristics (Table 1) are still met.

**Table 1:** Key changes between 2021 and 2014 TPPs for peripheral TB drug-susceptibility testing.

	2014		2021	
	Optimal	Minimal	Optimal	Minimal
**Goal**	Diagnosis of TB disease and detection of drug resistance to inform decision-making about the optimal first-line regimen (HRZE, REMox or PaMZ) for treatment, and possibly to detect the presence of additional resistance to second-line anti-TB agents and the need for further testing		Diagnosis of TB disease and detection of drug resistance to inform decision-making about the optimal (individualised) regimen	Diagnosis of TB disease and detection of drug resistance to provide rapid triage of patients and identification of adequate treatment regimen (first-line treatment versus second-line treatment)
**Priority drugs**	In order of decreasing importance: 1.RIF 2. FQs (including MFX) 3. INH and PZA (equally important) 4. AGs and CAP Optimally all drugs would be included, but as a minimum at least RIF should be included		In order of decreasing importance: Minimal + 1. PZA + LZD + Pa/DLM + CFZ 2. AMK + DCS 3. Any additional drug listed in the WHO treatment guidelines	RIF + INH+ FQ + BDQ (see explanation on BDQ in the main text) (FQ always includes LFX and MFX)
**Target population**	Target groups are all patients suspected of having TB, with a special focus on those at high risk of morbidity and mortality from drug-resistant TB, such as people living with HIV and those at high risk of having MDR-TB (for example, household contacts of patients diagnosed with MDR-TB, and persons with a history of TB, especially those for whom first-line therapy has failed) in countries with a medium prevalence to a high prevalence of TB as defined by WHO		People of all ages in need of evaluation for TB and those requiring drug resistance assessment	
**Sample type**	Unprocessed sputum		Unprocessed sputum and additional clinically relevant specimens for TB or other targeted diseases (see “Multi-use platform”)	Sputum and other clinically relevant specimens for TB, including (but not limited to) gastric aspirate, induced sputum, nasopharyngeal aspirate, and stool.

Abbreviations: AGs–aminoglycosides; AMK–amikacin; BDQ–bedaquiline; CAP–capreomycin; CFZ–clofazimine; DCS–D-cycloserine; DLM–delamanid; FQ–fluoroquinolone; HRZE–regimen of isoniazid, rifampicin, pyrazinamide, and ethambutol; INH–isoniazid; LFX–levofloxacin; LZD–linezolid; MDR-TB–multi-drug resistant TB; MFX–moxifloxacin; Pa–pretomanid; PaMZ–regimen of pretomanid, moxifloxacin, pyrazinamide; PZA–pyrazinamide; ReMOX–regimen of moxifloxacin, rifampicin, pyrazinamide and one of ethambutol or isoniazid; RIF–rifampicin; TB–tuberculosis; TPP–target product profile; WHO–World Health Organization.

### Ethical considerations

As this study was based on multiple rounds of questionnaires and discussion and no biological samples were required, the study was exempt from needing institutional review board approval.

## Results

### Participants

**Fig 1:**
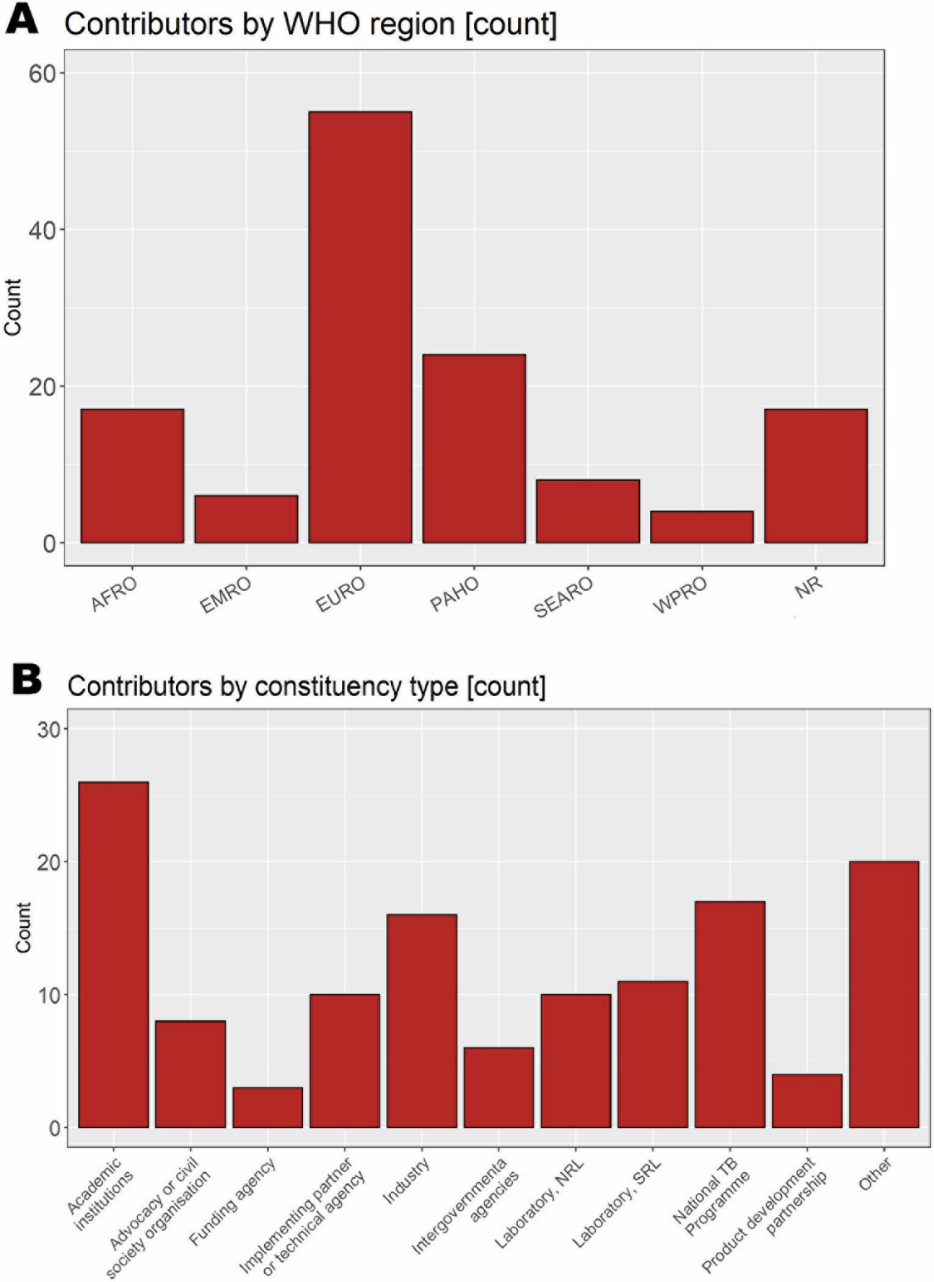
Participants in Delphi-like survey and call for public comment, grouped by **A)** World Health Organization region and **B)** Constituency type. AFRO–African Regional Office; EMRO–Eastern Mediterranean Regional Office; EURO–European Regional Office; NR–Not reported; NRL–National TB reference laboratory; PAHO–Pan-American Health Office; SEARO–South-East Asian Regional Office; SRL–Supranational TB reference laboratory; TB–tuberculosis; WPRO–Western Pacific Regional Office.

The TPP document and survey were responded to by 46 individuals in the Delphi-like process, while the call for public comment was responded to by 85 individuals. The largest proportion of contributors to the Delphi-like process and call for public comment were from the WHO European Region (42%, 55/131), with the smallest proportion from Western Pacific Region (3%, 4/131) (Fig 1A). Respondents to the Delphi survey and public comment represented various constituencies (Fig 1B). Academic researchers (20%, 26/131, NTPs (13%, 17/131), and industry (12%, 16/131) participated in high numbers, with stakeholders from implementation partners, the laboratory sectors, and civil society groups also well-represented. There were almost 70 stakeholders from 25 countries at the WHO virtual consultation throughout its four days.

### Changes to the TPP through the consensus-building process

The working document first circulated for comment in a Delphi-like process was broadly accepted by the expert group surveyed. Notably, agreement was reached for 42/47 (89%) components of the document. Exceptions included characteristics related to the prioritisation of anti-TB agents targeted by the assays for resistance detection, pricing, and performance characteristics. After revision and circulation for public comment, the broader audience continued to express some disagreement with revised characteristics pertaining to the prioritisation of anti-TB agents, test pricing, the target population of the updated TPP, and time-to-result.

### Primary updates to a TPP for DST at peripheral centres

The overall aim of the TPP is to describe the desired characteristics of assays that would detect *Mycobacterium tuberculosis* (MTB) and provide antimicrobial susceptibility testing results at peripheral centres to inform timely anti-TB treatment decisions, with special consideration given to new TB treatment regimens. Notable and important changes in the 2021 TPP [16] compared to the 2014 TPP [17] were made to reflect current diagnostic needs and technology advancements, and concern characteristics describing goals, priority drugs, target population, and sample type (Table 1). The remaining characteristics were updated to mirror these fundamental updates [16].

New priority guidance highlights the need to move from assays mainly focused on first-line drugs to more comprehensive solutions to inform optimal, individualised regimens for second-line treatment.

With updated treatment regimens now recommended by WHO, the TPP was altered to reflect these new realities (including the updated definitions of pre-XDR and XDR-TB) [15, 18]. First, the priority of anti-TB agent resistance testing now includes BDQ and INH as Minimal criteria, joining RIF and FQ (which always includes moxifloxacin [MFX] and levofloxacin [LFX]). This replaces the previous Priority Drugs Minimal criteria of RIF- and MFX-resistance testing, which would have informed then-current optimal first-line treatment regimens (i.e., HRZE [regimen of isoniazid, rifampicin, pyrazinamide, and ethambutol], REMox [regimen of moxifloxacin, rifampicin, pyrazinamide and one of ethambutol or isoniazid], or PaMZ [regimen of pretomanid, moxifloxacin, pyrazinamide]). The inclusion of INH enables improved treatment decisions for patients with RIF-susceptible and INH-resistant TB, which is largely missed but requires appropriate treatment [15]. Because BDQ has now replaced the injectable agents in all MDR/RR-TB regimens and is used in pre-XDR-TB regimens [15], it was judged important enough to include as a Minimal target. It should be noted that while BDQ is a high priority drug for treatment, resistance is still low globally and relevant mutations associated with resistance have yet to be fully elucidated. It thus remains an aspirational criterion currently for molecular-based testing, but is expected to be attainable in three to five years. Along with drugs listed under Minimal priority, the Optimal priority of drugs reflects the decreased importance or outright recommendation against the use of specific second-line injectable agents [15], and the increased prominence of pyrazinamide (PZA), LZD, and clofazimine (CFZ) in treating drug-resistant forms of TB. PZA has enabled the treatment of drug-susceptible TB to be shortened from nine to six months and plays an important role in treating RR/MDR-TB if effective regimens cannot be otherwise constructed (e.g., groups A and B drugs unavailable). However, since PZA resistance is significantly associated with RIF resistance, PZA should only be used to treat RR/MDR-TB if susceptibility is first confirmed [19]. Therefore, determining PZA susceptibility patterns accurately is crucial to designing appropriate treatments. LZD (a group A drug together with FQ and BDQ, thus relevant for defining XDR-TB), can increase treatment effectiveness if used for at least six months; however, given the toxicity potentially limiting its use the drug was included among the Optimal requirement. Additional drugs belonging groups B and C follow in decreasing order of priority: pretomanid (Pa)/delamanid (DLM) + CFZ, AMK + DCS.

Another important update under the scope of the TPP is that the target population has been made more inclusive. All individuals of any age who require evaluation for TB are included under a common Minimal and Optimal requirement for the characteristic Target population. This brings the TPP in line with the End TB Strategy’s call for universal DST for all individuals undergoing TB testing [20]. The Sample Type characteristic has been updated to stress that new assays should be able at Minimum to test other processed or unprocessed specimens besides sputum, with additional unprocessed clinically relevant specimens given as Optimal requirements. Accordingly, the 2021 TPP includes consideration that assay performance should be independent of interfering substances. Relevant specimens for extrapulmonary TB should also be considered. The revision of these characteristics reflects the reality and difficulty in obtaining sputum samples from specific populations, including children and people living with HIV, and the growing evidence base for non-sputum samples, including tongue swabs and aerosol capture for molecular diagnostics. The updated TPP also highlights the desire for a multi-use platform (previously not considered among Minimal requirements). Indeed, the possibility of implementing multi-disease solutions at primary healthcare centres would greatly improve triage and access to treatment for people affected by TB. It could be particularly relevant for diseases presenting with overlapping symptoms with TB, as recently demonstrated during the COVID-19 pandemic [21]. In that vein, the TPP was worded to encourage movement away from current sputum-based diagnostics and emphasises that the assay defined by the TPP should be scalable at peripheral centres. Therefore, point-of-care tests are now included as an Optimal requirement and reflect technology changes that appear to be bringing these approaches within reach.

### Secondary updates

In general, further updates have been driven by the need to align the document with the current state-of-art or new technologies entering the diagnostics market.

### Performances characteristics

Performances of the assay defined in the document for MTB detection should not be inferior to current available rapid diagnostic assays (Minimal: limit of detection ≤10^4^ CFU/mL, sensitivity on smear-negative >60%, sensitivity on smear-positive ≥99%, specificity ≥98%). Whereas specificity for DST should be >98% against both genotypic (whole genome/targeted sequencing) and phenotypic (culture-based) reference standards, sensitivity for DST compared with phenotypic DST as reference consider current knowledge on the genetic determinants of drug resistance to new and repurposed drugs (Minimal: RIF >95%; INH, FQ >90%; BDQ, LZD, CFZ, DLM, pretomanid, AMK, PZA ≥80%). Considering continuous updates and increasing understanding of genotypic-phenotypic associations, new assays should aim at >95% sensitivity for all the drugs considered (Optimal requirement). Sensitivity for DST against a genotypic reference standard should be >98% to guarantee accurate detection of genetic mutations.

Under ideal conditions, phenotypic DST can be used to detect resistance when 1% or more of the bacterial population is drug-resistant, except in the case of PZA [22]. However, the limit of detection of rapid genotypic assays to detect minor populations of resistant mutants greatly depends upon the method used [23]. New technologies (e.g., next-generation sequencing (NGS)) may reach the sensitivity of culture-based DST. For this reason, a limit of detection of minor variants has been included as a new characteristic for the assay described in the TPP (Minimal ≤20%; Optimal ≤3%).

Another important update to the TPP is the Minimal requirements for the Time to result characteristic. At Minimal, a new test should produce detection and DST results in under 6 hours to allow next-day treatment decisions. Optimally, these same results should be available in under 30 minutes to permit same-day treatment decisions. While a short time-to-result is important to reduce pretreatment loss to follow-up [24], compared to the 2014 TPP [17], these Minimal and Optimal durations have been increased to reflect the reality of sequencing technologies. Next-generation sequencing is being increasingly rolled out [25], and the TPP must balance what is technologically possible in this space with realities in peripheral healthcare settings, particularly since sequencing may be the only option for the detection of mutations that are known to arise across the entire sequence of genes associated with drug-resistance.

### Operational characteristics

Directly linked to Time to result, Daily throughput and Walk-away operation characteristics are slightly revised. Regarding Daily throughput, new tests should Minimally run at least 10 tests a day, and Optimally should run at least 25 tests a day; 10 tests was chosen as a cut-off as most peripheral centres will not regularly need to run more tests than that. A Sample capacity and throughput characteristic is included to indicate that batching of samples should be possible and that the running of one specimen on the instrument should not preclude starting another run. The Walk-away operation characteristic indicates that no more than one step of operator intervention should be needed once the sample has been placed into the system. All of these characteristics aim to improve the simplicity of laboratory workflows and increase the number of samples that can be handled each day. As outlined for the time-to-result characteristic, the Manual preparation of samples intended as the number of steps needed after obtaining a sample has been slightly increased (Minimal ≤ 5 steps *vs* ≤ 2 steps in 2014) to reflect the reality of some currently innovative technologies.

Biosafety and Waste disposal characteristics for the assay described in the TPP document have been aligned with current requirements for low-risk TB laboratories and WHO-endorsed TB assays at the peripheral level. A call for reusable, recyclable or non-plastic alternatives to disposable materials is also included. Additional operational characteristics (Instrument, Power requirements, Maintenance and calibration, Data export and analysis, Reagent transport and storage, Training and education) have been marginally revised to improve clarity and highlight some critical aspects encountered during the roll-out of new molecular assays at peripheral levels in the past few years. Particular attention has been paid to environmental conditions (dust, humidity, temperature) and the possibility of providing remote assistance for maintenance/calibration, data handling, and training.

### Pricing of tests and instruments

For assays able to detect resistance to the highest priority anti TB agents identified as part of the Minimal characteristics (RIF, INH, FQ), the price has been set at 10–15 USD under the Minimal scenario, while under the Optimal scenario the maximum price for this combination would be 5 USD. Pricing for BDQ requires additional consideration because resistance testing for this drug would likely require add-on or different technology, and the molecular basis of BDQ-resistance has yet to be fully elucidated. It was not possible at this time to set pricing requirements for assays able to target the highest priority anti TB agents indicated under the Optimal scenario, as there are no data regarding the cost of covering new drugs like DLM, LZD, and CFZ. However, it should be noted that the price of the new Xpert XDR-TB assay, which tests for resistance to INH, FQ, second-line injectables, and ethionamide, is around 20 USD [26]: a price much higher than this would have to be justified by significant added value in terms of accuracy, deployability in peripheral locations, and the array of anti-TB agents which it can test.

Capital costs for the instrument have been updated to Minimal less than 20,000 USD and Optimally less than 5000 USD, with warranties, services contracts, and technical support for 3 years included in both conditions. Lower capital costs would decrease a significant barrier to their implementation in resource-limited settings, which should lead to an increase in DST rates. Manufacturers should consider novel acquisition models, such as reagent rental or a cost-per-result model to improve affordability of instruments for low- and middle-income countries.

## Discussion

The 2014 TPPs [12] have set requirements for specific use-cases of new diagnostic tests and served to inform the development of several new TB diagnostics such as Xpert XDR and molecular tests for PZA since their publication. It is now common to see diagnostic accuracy studies of new tools, such as biomarkers [27, 28], transcriptional signatures [29, 30], or artificial intelligence-aided chest radiographs [31] reported and discussed with respect to their relevant TPP. In this way, it is evident that TPPs serve as platforms to aid communication between various stakeholders by laying out a set of criteria.

Meeting the End TB goals will require further scale-up of current tools, as well as innovative new tools to improve the quality of TB care and further reach people with TB in peripheral settings when they first present. With aspirational Optimal characteristics specified to reflect ‘best-case scenarios’, the TPP’s Minimal characteristics have been specified with the realities and needs of peripheral healthcare centres in mind, particularly in low- and middle-income countries (LMICs), providing pragmatic product requirements for developers. To ensure these novel DST tools will be impactful, they must be designed for use in and be accessible to LMICs; once a product is developed, operational research to understand logistical issues, barriers to use, and guide implementation will be necessary [32]. This updated TPP is intended to guide development of products that will lead to increased availability to TB and DST testing through simplified, user-friendly assays that are affordable, accurate, and reliable in peripheral settings. Recent advances in technology combined with the characteristics specified in the new TPP are expected to facilitate the rapid development of high-priority products [33].

Developing accurate molecular tests for drug resistance relies on knowing the mutations associated with drug resistance that should be targeted. As previously mentioned, for newer or repurposed drugs like BDQ or CFZ, WHO-endorsed molecular assays are not yet available, but standard culture-based phenotypic testing capabilities also remain limited [34]. Furthermore, interpretation of the genotype-phenotype associations is hampered by current gaps in our understanding of the genetic basis of resistance. The WHO published its first catalogue of mutations and their association with resistance to address this need and facilitate research and development [35, 36]. Regular updates are planned, and improvements to the catalogue are expected as more resistant isolates are collected, especially for new and repurposed drugs. Appropriate classification of borderline mutations is another knowledge gap. Research including in vivo and in vitro selection experiments and on the association between mutations (or combinations of mutations) and minimum inhibitory concentrations is needed to provide clarity [37].

TPPs are a useful starting point for a longer product development process for product developers and researchers seeking to bring new tests to market. The total cost of implementing a product is another important factor influencing uptake, and cost-effectiveness analyses to understand the added value while considering the willingness to pay thresholds will be helpful. Lastly, it is important to note that TPPs are a guide. A product that does not fulfil all Minimal requirements but offers important public health benefits could still be considered for WHO policy recommendations.

## Conclusions

Updating the TPP for DST in peripheral settings was a collaborative effort that gathered extensive inputs from the TB community and a broad coalition of stakeholders. The TPP serves to guide research and development of new products to address the greatest expected needs in TB DST in the upcoming five years. The revised document is aligned with the latest WHO definitions of drug resistance and treatment regimens, while also considering the future landscape. Advances in diagnostic technologies have progressed substantially in the past several years and have been particularly accelerated by efforts in combatting SARS-CoV-2; these development could represent an opportunity for novel TB assays. The TPP is now more patient-centred and inclusive, considering different types of TB, target populations, and a broader array of sample types. Achieving the ambitious target of ending TB by 2035 will require significant, sustained investments in diagnostic tools that can maximally impact the patient pathway to deliver rapid, accurate, and quality-assured results to inform timely treatment decisions [38].

## Supporting information

S1 File Questions included in public consultation survey. This file contains the fields included in the public comment survey sent out during the development process of the updated TPP for Next-Generation DST at Peripheral Centres.
(XLSX)

## References

[1] World Health Organization. Global Tuberculosis Report 2021. Geneva: World Health Organization, 2021.

[2] Subbaraman R, Jhaveri T, Nathavitharana RR. Closing gaps in the tuberculosis care cascade: an action-oriented research agenda. J Clin Tuberc Other Mycobact Dis. 2020;19:100144–. doi: 10.1016/j.jctube.2020.100144 32072022 PMC7015982

[3] Pai M, Schito M. Tuberculosis Diagnostics in 2015: Landscape, Priorities, Needs, and Prospects. Journal of Infectious Diseases. 2015;211(suppl 2):S21–S8. doi: 10.1093/infdis/jiu803 25765103 PMC4366576

[4] World Health Organization. WHO consolidated guidelines on tuberculosis. Module 3: Rapid diagnostics for tuberculosis detection. 2021 Update. Geneva: World Health Organization, 2021.

[5] Theron G, Zijenah L, Chanda D, Clowes P, Rachow A, Lesosky M, et al. Feasibility, accuracy, and clinical effect of point-of-care Xpert MTB/RIF testing for tuberculosis in primary-care settings in Africa: a multicentre, randomised, controlled trial. Lancet. 2014;383(9915):424–35. Epub 2013/11/02. doi: 10.1016/S0140-6736(13)62073-5 24176144

[6] World Health Organization. Meeting report of the WHO expert consultation on the definition of extensively drug-resistant tuberculosis, 27–29 October 2020. Geneva: World Health Organization, 2021.

[7] Meehan CJ, Goig GA, Kohl TA, Verboven L, Dippenaar A, Ezewudo M, et al. Whole genome sequencing of Mycobacterium tuberculosis: current standards and open issues. Nature reviews Microbiology. 2019;17(9):533–45. Epub 2019/06/19. doi: 10.1038/s41579-019-0214-5 31209399

[8] Jouet A, Gaudin C, Badalato N, Allix-Béguec C, Duthoy S, Ferré A, et al. Deep amplicon sequencing for culture-free prediction of susceptibility or resistance to 13 anti-tuberculous drugs. The European respiratory journal. 2021;57(3). Epub 2020/09/19. doi: 10.1183/13993003.02338-2020 32943401 PMC8174722

[9] Kohli M, MacLean E, Pai M, Schumacher SG, Denkinger CM. Diagnostic accuracy of centralised assays for TB detection and detection of resistance to rifampicin and isoniazid: a systematic review and meta-analysis. The European respiratory journal. 2021;57(2). Epub 2020/08/29. doi: 10.1183/13993003.00747-2020 32855226

[10] Treatment Action Group. 2021 Pipeline Report: Tuberculosis Diagnostics. New York: Treatment Action Group, 2021 November 2021. Report No.

[11] FIND. Dx Pipeline Status: Next-generation drug susceptibility testing at microscopy centres [Webpage]. Geneva: FIND; 2022 [updated 2020; cited 2022 20 June]. Available from: https://www.finddx.org/dx-pipeline-status/.

[12] World Health Organization. High-priority target product profiles for new tuberculosis diagnostics: report of a consensus meeting. Geneva: World Health Organization, 2014 29 April 2014.

[13] MacLean E, Kohli M, Weber SF, Suresh A, Schumacher SG, Denkinger CM, et al. Advances in Molecular Diagnosis of Tuberculosis. J Clin Microbiol. 2020;58(10). Epub 2020/08/08. doi: 10.1128/JCM.01582-19 32759357 PMC7512154

[14] Tiberi S, du Plessis N, Walzl G, Vjecha MJ, Rao M, Ntoumi F, et al. Tuberculosis: progress and advances in development of new drugs, treatment regimens, and host-directed therapies. Lancet Infect Dis. 2018;18(7):e183–e98. Epub 2018/03/28. doi: 10.1016/S1473-3099(18)30110-5 29580819

[15] World Health Organization. WHO consolidated guidelines on tuberculosis: Module 4: Treatment: drug-resistant tuberculosis treatment. Geneva: World Health Organization, 2020. 32603040

[16] World Health Organization. Target product profile for next-generation drug-susceptibility testing at peripheral centres. Geneva: World Health Organization, 2021.

[17] World Health Organization. Meeting report: High-priority target product profiles for new tuberculosis diagnostics: report of a consensus meeting. Next-generation drug-susceptibility testing at microscopy centres. Geneva: World Health Organization; 2014. p. 34–46.

[18] World Health Organization. WHO consolidated guidelines on tuberculosis. Module 1: Prevention–tuberculosis preventive treatment. Geneva: World Health Organization, 2020. 32186832

[19] Zignol M, Dean AS, Alikhanova N, Andres S, Cabibbe AM, Cirillo DM, et al. Population-based resistance of Mycobacterium tuberculosis isolates to pyrazinamide and fluoroquinolones: results from a multicountry surveillance project. Lancet Infect Dis. 2016;16(10):1185–92. Epub 2016/07/12. doi: 10.1016/S1473-3099(16)30190-6 27397590 PMC5030278

[20] World Health Organization. WHO End TB Strategy. Geneva: World Health Organization; 2015.

[21] Mohr-Holland E, Hacking D, Daniels J, Scott V, Mudaly V, Furin J, et al. Diagnosis patterns for rifampicin-resistant TB after onset of COVID-19. The International Journal of Tuberculosis and Lung Disease. 2021;25(9):772–5. doi: 10.5588/ijtld.21.0340 34802503 PMC8412107

[22] Canetti G, Fox W, Khomenko A, Mahler HT, Menon NK, Mitchison DA, et al. Advances in techniques of testing mycobacterial drug sensitivity, and the use of sensitivity tests in tuberculosis control programmes. Bulletin of the World Health Organization. 1969;41(1):21–43. Epub 1969/01/01. 5309084 PMC2427409

[23] Rigouts L, Miotto P, Schats M, Lempens P, Cabibbe AM, Galbiati S, et al. Fluoroquinolone heteroresistance in Mycobacterium tuberculosis: detection by genotypic and phenotypic assays in experimentally mixed populations. Scientific reports. 2019;9(1):11760. Epub 2019/08/15. doi: 10.1038/s41598-019-48289-9 31409849 PMC6692311

[24] Subbaraman R, Nathavitharana RR, Satyanarayana S, Pai M, Thomas BE, Chadha VK, et al. The Tuberculosis Cascade of Care in India’s Public Sector: A Systematic Review and Meta-analysis. PLoS Med. 2016;13(10):e1002149. Epub 2016/10/26. doi: 10.1371/journal.pmed.1002149 27780217 PMC5079571

[25] Cohen KA, Manson AL, Desjardins CA, Abeel T, Earl AM. Deciphering drug resistance in Mycobacterium tuberculosis using whole-genome sequencing: progress, promise, and challenges. Genome medicine. 2019;11(1):45. Epub 2019/07/28. doi: 10.1186/s13073-019-0660-8 31345251 PMC6657377

[26] Stop TB Partnership. Global Drug Facility: Diagnostics Catalogue. Geneva: Stop TB Partnership, 2021.

[27] Moreira FMF, Verma R, Pereira Dos Santos PC, Leite A, da Silva Santos A, de Araujo RCP, et al. Blood-based host biomarker diagnostics in active case finding for pulmonary tuberculosis: A diagnostic case-control study. EClinicalMedicine. 2021;33:100776. Epub 2021/04/13. doi: 10.1016/j.eclinm.2021.100776 33842866 PMC8020164

[28] MacLean E, Broger T, Yerlikaya S, Fernandez-Carballo BL, Pai M, Denkinger CM. A systematic review of biomarkers to detect active tuberculosis. Nat Microbiol. 2019;4(5):748–58. Epub 2019/02/26. doi: 10.1038/s41564-019-0380-2 30804546

[29] Scriba TJ, Fiore-Gartland A, Penn-Nicholson A, Mulenga H, Kimbung Mbandi S, Borate B, et al. Biomarker-guided tuberculosis preventive therapy (CORTIS): a randomised controlled trial. Lancet Infect Dis. 2021. Epub 2021/01/29. doi: 10.1016/s1473-3099(20)30914-2 33508224 PMC7907670

[30] Turner CT, Gupta RK, Tsaliki E, Roe JK, Mondal P, Nyawo GR, et al. Blood transcriptional biomarkers for active pulmonary tuberculosis in a high-burden setting: a prospective, observational, diagnostic accuracy study. Lancet Respir Med. 2020;8(4):407–19. Epub 2020/03/18. doi: 10.1016/S2213-2600(19)30469-2 32178775 PMC7113842

[31] Qin ZZ, Ahmed S, Sarker MS, Paul K, Adel ASS, Naheyan T, et al. Tuberculosis detection from chest x-rays for triaging in a high tuberculosis-burden setting: an evaluation of five artificial intelligence algorithms. The Lancet Digital health. 2021;3(9):e543–e54. Epub 2021/08/28. doi: 10.1016/S2589-7500(21)00116-3 34446265

[32] World Health Organization. Policy framework: Implementing tuberculosis diagnostics. Geneva: World Health Organization, 2015.

[33] Kik SV, Denkinger CM, Casenghi M, Vadnais C, Pai M. Tuberculosis diagnostics: which target product profiles should be prioritised? The European respiratory journal. 2014;44(2):537–40. Epub 2014/04/04. doi: 10.1183/09031936.00027714 24696110

[34] Farooq HZ, Cirillo DM, Hillemann D, Wyllie D, van der Werf MJ, Ködmön C, et al. Limited Capability for Testing Mycobacterium tuberculosis for Susceptibility to New Drugs. Emerging infectious diseases. 2021;27(3):985–7. Epub 2021/02/25. doi: 10.3201/eid2703.204418 33622487 PMC7920658

[35] World Health Organization. Catalogue of mutations in Mycobacterium tuberculosis complex and their association with drug resistance. Geneva: World Health Organization, 2021.

[36] Walker TM, Miotto P, Köser CU, Fowler PW, Knaggs J, Iqbal Z, et al. The 2021 WHO catalogue of Mycobacterium tuberculosis complex mutations associated with drug resistance: A genotypic analysis. The Lancet Microbe. 2022;3(4):e265–e73. Epub 2022/04/05. doi: 10.1016/S2666-5247(21)00301-3 35373160 PMC7612554

[37] World Health Organization. Optimized broth microdilution plate methodology for drug susceptibility testing of Mycobacterium tuberculosis complex. Geneva: World Health Organization, Programme GT; 2022 22 April 2022.

[38] Fleming KA, Horton S, Wilson ML, Atun R, DeStigter K, Flanigan J, et al. The Lancet Commission on diagnostics: transforming access to diagnostics. Lancet. 2021;398(10315):1997–2050. Epub 2021/10/10. doi: 10.1016/S0140-6736(21)00673-5 34626542 PMC8494468

